# Neuroleptic Malignant Syndrome Following Brexpiprazole Initiation in a Treatment-Resistant Schizophrenia Patient With Influenza A: A Case Report

**DOI:** 10.7759/cureus.83087

**Published:** 2025-04-27

**Authors:** Kar Yin Lee, Shze Yee Choo, Chao Tian Tang

**Affiliations:** 1 Department of Psychiatry, Sengkang General Hospital, Singapore, SGP

**Keywords:** antipsychotics, brexpiprazole, influenza a, influenza vaccination, neuroleptic malignant syndrome, treatment-resistant schizophrenia

## Abstract

We present a case of a 30-year-old male with a 15-year history of treatment-resistant schizophrenia who developed neuroleptic malignant syndrome (NMS) after the initiation of brexpiprazole in the setting of serology-confirmed influenza A infection. The patient was also receiving maintenance electroconvulsive therapy (ECT) and was undergoing cross-titration of antipsychotics when he developed fever, cough, shortness of breath, tremors, and rigidity. He tested positive for influenza A, and there were no records of any regular influenza vaccination. This case underscores the need for caution in the initiation of antipsychotics, including brexpiprazole, in this group of patients and the potential role of systemic infections in precipitating NMS. Early recognition and prompt intervention are crucial in preventing morbidity and mortality. Furthermore, this case highlights the importance of regular influenza vaccination, particularly in individuals with serious mental illness and on long-term antipsychotic therapy.

## Introduction

Neuroleptic malignant syndrome (NMS) is a potentially fatal idiosyncratic reaction to dopamine-blocking agents, characterized by hyperthermia, autonomic instability, altered mental status, and rigidity [1]. According to the Diagnostic and Statistical Manual of Mental Disorders, Fifth Edition, Text Revision (DSM-5-TR) diagnostic criteria, NMS is classified as a sub-category of medication-induced movement disorders [2]. Although the underlying pathophysiology is complex and remains incompletely understood, several mechanisms have been proposed. The commonly recognized theories are neuroleptic‐induced alteration of central neuroregulatory mechanisms. The blockade of dopamine D2 receptor or abrupt withdrawal of dopamine D2 receptor stimulation is believed to disrupt dopaminergic pathways in the brain, leading to hallmark symptoms of NMS like muscle rigidity and autonomic dysfunction [3]. Another proposed mechanism involves increased sympathetic nervous system activity. The blockade of dopamine receptors may lead to a compensatory increase in sympathetic tone, resulting in autonomic instability [4]. Genetic predisposition may also play a role in the development of NMS. Some individuals may have a genetic susceptibility that predisposes them to this condition, although specific genetic markers have not been conclusively identified [5].

NMS is more commonly associated with first-generation antipsychotics due to their greater dopamine D2 receptor antagonism. However, NMS has been reported with second-generation antipsychotics. Second-generation antipsychotics like brexpiprazole were approved by the Food and Drug Administration (FDA) for the treatment of schizophrenia [6]. The efficacy and safety of brexpiprazole were assessed in several published placebo-controlled clinical trials, including Kane et al. [7] and Correll et al. [8]. The most common side effects of brexpiprazole include akathisia and weight gain, with additional warning for NMS, which is similar to other second-generation antipsychotics. Although NMS is a rare complication of antipsychotic therapy, it can potentially lead to serious and fatal outcomes. Incidence rates for NMS range from 0.02% to 3% among patients taking antipsychotic agents [3,9]. Early reports indicated mortality rates exceeding 30% for NMS; however, with increased awareness, earlier detection, and improved supportive care, these rates have declined to less than 10% [10-12].

Some common risk factors for NMS include concomitant use of other psychotropic drugs or lithium, higher-potency antipsychotics, rapid dose escalation, depot formulations, comorbid substance use, neurologic disease, and acute medical illness, including infection [9,13-16]. Systemic infections, such as influenza A, may act as potential stressors that exacerbate or trigger NMS. Recent literature suggests that infections can precipitate NMS by inducing systemic inflammation, metabolic stress, and increased cytokine activity, leading to dopaminergic dysregulation [17]. Furthermore, patients with schizophrenia have an elevated risk of severe infections due to metabolic syndrome, immunomodulatory effects of antipsychotics, and barriers to accessing healthcare [18]. Despite these risks, vaccination rates in individuals with mental illness remain lower than in the general population, raising concerns about preventable morbidity [19].

We report a case of NMS in a patient with treatment-resistant schizophrenia following the initiation of brexpiprazole, in the context of serology-confirmed influenza A infection. This case report aims to highlight the diagnostic challenges and therapeutic considerations in such a scenario, as well as the potential protective role of influenza vaccination in patients receiving long-term antipsychotic therapy.

## Case presentation

The patient is a 30-year-old male who was diagnosed with schizophrenia at the age of 15, presenting with auditory hallucinations, persecutory delusions, and aggressive behavior toward family members. He has tried several antipsychotics, including haloperidol, olanzapine, risperidone, and amisulpride, but with only partial therapeutic response. His diagnosis was revised to treatment-resistant schizophrenia at the age of 20 due to the poor response to treatment. Clozapine and maintenance electroconvulsive therapy (ECT) were introduced as part of his treatment regimen. Although he had a history of extrapyramidal side (EPS) effects, he had never experienced NMS, even during previous psychotropics adjustments. He had never received depot anti-psychotics, and there was no history of illicit drug or alcohol use.

Prior to the febrile episode, he had been admitted to a mental health institution for management of treatment-resistant schizophrenia. He was maintained on clozapine 200 mg twice daily, aripiprazole 30 mg once in the morning, mirtazapine 15mg once at night, sodium valproate 500 mg once at night, and memantine 15 mg once at night. He was on this regimen of multiple psychotropics as adjunctive treatment to clozapine for treatment-resistant schizophrenia. On the day before the onset of fever, he underwent cross-titration from aripiprazole to brexpiprazole, with aripiprazole reduced from 30 mg to 15 mg once in the morning and brexpiprazole initiated at 1 mg once in the morning. Later that same day, he developed flu-like symptoms including low-grade fever, cough, and shortness of breath. Consequently, he was transferred from the mental health institution to a general medical hospital for further investigation and treatment. He was admitted to a general medicine ward, where the primary medical team consulted an in-house psychiatrist and neurologist for possible NMS.

His condition deteriorated rapidly in the next 48 hours with progressive confusion, diaphoresis, autonomic instability (tachycardia, labile blood pressure), tremors, and muscle rigidity. His body temperature was 37.5°C on admission and peaked at 40.5°C on the third day of hospitalization. In view of concurrent upper respiratory tract infectious symptoms, the respiratory swab was done, and he tested positive for influenza A. He was started on oseltamivir (Tamiflu). He had not received regular influenza vaccinations, based on the records. Other investigations revealed markedly elevated creatine kinase (CK) levels, peaking at 29,591 U/L on admission, along with leukocytosis, metabolic acidosis, and elevated liver enzymes. All psychotropic medications were immediately withheld on admission for possible NMS. Other differential diagnoses considered were influenza-related rhabdomyolysis, viral encephalitis, and serotonin syndrome. Physical examination revealed no hyperreflexia, ataxia, or myoclonus, which made serotonin syndrome less likely. Given his persistent altered mental status and febrile illness, extensive investigations, including cerebrospinal fluid (CSF) analysis and neuroimaging, including CT brain and a contrasted MRI brain, were performed to exclude intracranial infections. CSF analysis was not suggestive of other intracranial causes of his presentation, and the rest of the investigations were normal. He was managed with aggressive intravenous hydration, benzodiazepines, and bromocriptine with intensive monitoring for NMS.

**Figure 1:**
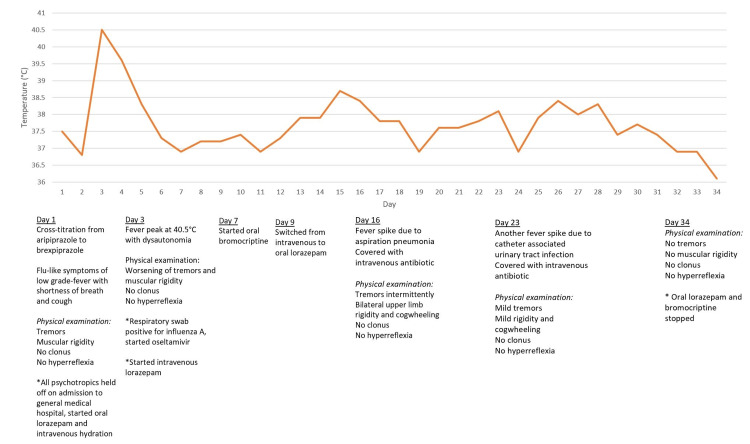
Fever trend and timeline

**Figure 2:**
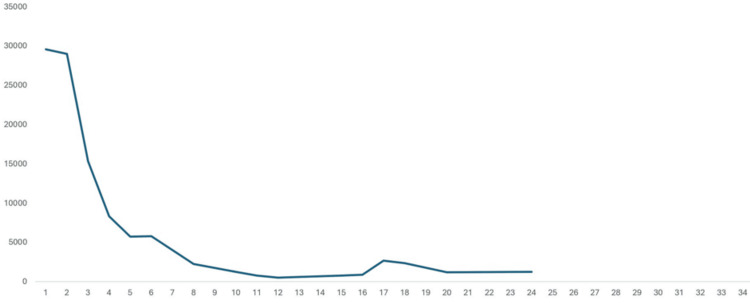
Creatinine kinase trend (U/L)

Over the course of two weeks, he developed aspiration pneumonia and catheter-associated urinary tract infection, which he was successfully treated with antibiotics. Over the course of another two weeks, the patient demonstrated gradual clinical improvement, with resolution of autonomic instability and normalization of CK levels. Muscle rigidity and tremors showed significant improvement. Bromocriptine and lorazepam were stopped after the complete resolution of rigidity. Following the resolution of his NMS and infections, the patient was transferred back to the mental health institute. Clozapine was reintroduced, and ECT was also resumed. There were no further episodes of NMS following the re-initiation of clozapine. Figure 1 illustrates the fever trend and timeline of events, and Figure 2 charts the creatinine kinase trend.

## Discussion

To our knowledge, this is the first case report of NMS while on cross-titration from aripiprazole to brexpiprazole, in the setting of serology-confirmed influenza A. NMS is more commonly reported with the use of dopamine-receptor antagonist medications or with rapid withdrawal of dopaminergic medications [20]. It is not typical of NMS occurrence during gradual cross-titration between two partial dopamine agonists, as reported in this case. Brexpiprazole is a partial agonist for the D2 and 5-HT1A receptors, and it has a complex spectrum of agonism and antagonism for other 5-HT receptors [21]. Aripiprazole is a quinolinone antipsychotic that is a partial agonist at the D2 and 5HT-1A receptors and an antagonist at the 5HT-2A receptor [22]. Both are atypical antipsychotics and FDA-approved for schizophrenia [21]. A review of literature via several databases including PubMed, Google Scholar and Scopus did not turn out any literature or case reports on patients who have experienced NMS, which has been induced by brexpiprazole with comorbid serology-confirmed influenza A. Gautam et al. [23] presented a case where possible NMS occurred in the setting of brexpiprazole discontinuation and initiation on olanzapine which was subsequently cross tapered to quetiapine and finally haloperidol on the background of possible cannabinoid uses which was rapidly reversed by intravenous Lorazepam which was not done in our case. This case highlights the need for clinicians to be vigilant in monitoring rarer complications such as NMS, even with medications that have been thought to have a lower propensity for NMS, which have not yet been reported in the literature.

NMS remains a critical and potentially life-threatening condition, particularly in individuals with schizophrenia receiving antipsychotic treatment. Risk factors for NMS in this case included the concurrent use of multiple psychotropic medications and the presence of a systemic infection. Concurrent systemic infections, such as influenza A in this case, could be a red herring in unmasking NMS, in addition to precipitating or exacerbating NMS. The inflammatory response and metabolic stress associated with infections may disrupt central dopaminergic pathways, thereby increasing susceptibility to NMS [17]. Although specific cases linking influenza A to NMS are limited, the role of infections as potential triggers for NMS has been acknowledged in the literature [24]. The presence of an infection may initially divert clinical attention away from NMS, especially when leukocytosis and elevated inflammatory markers are attributed to infection rather than autonomic instability or muscle breakdown. Additionally, overlapping symptoms of infection, such as fever, tachycardia, and altered mental status, can closely resemble those of NMS, leading to diagnostic uncertainty and delays. Before initiating, dose adjusting, or cross-titrating antipsychotics, clinicians should assess for underlying infections, as symptoms like fever, respiratory distress, and confusion may overlap with those of NMS. If an infection is identified, appropriate management should precede any adjustments in antipsychotic therapy. A high index of suspicion is essential to distinguish infection-related manifestations from NMS, facilitating timely diagnosis and intervention.

Lastly, the absence of influenza vaccination may have increased the patient's susceptibility to viral infection, acting as a physiological stressor that potentially increases the risk of NMS in this case [19]. Influenza vaccination is a crucial preventive measure, particularly in individuals on long-term antipsychotic therapy, who may have a heightened risk of infections due to immunomodulatory effects of psychotropics, metabolic dysregulation, and psychiatric illness-related healthcare disparities. Patients with mental illness have been found to have higher rates of infection-related morbidity and mortality, and influenza infections in such patients may lead to severe systemic complications [25], including NMS. Despite so, the rate of influenza vaccination remains low in those who have mental illness compared to the general population. The World Health Organization (WHO) has estimated that global seasonal influenza vaccination coverage among older adults is approximately 50%, but this figure masks significant disparities between regions [26]. Studies in clinical cohorts with severe mental illness have found influenza vaccination rates from 25% [27] to as low as 7% [28]. As demonstrated in this case, the absence of influenza vaccination may have increased the patient's susceptibility to influenza, which in turn acted as a physiological stressor, potentially contributing to the development of NMS. Given these risks, regular tracking and monitoring of a patient’s influenza vaccination status should be considered for patients receiving long-term antipsychotic therapy to reduce the risk of systemic infections and to potentially mitigate a confluence of factors that can predispose to life-threatening complications such as NMS.

## Conclusions

Clinicians should maintain a high index of suspicion for NMS in patients presenting with changes in mental status and autonomic instability, especially in the setting of polypharmacy and systemic infections such as influenza A. While the case discussed presents an uncommon confluence of variables, it highlights the need for vigilance as well when using newer antipsychotics, including brexpiprazole, where NMS is not commonly expected to occur. Recent medication adjustments, including cross-titration of medications, and the presence of concurrent medical conditions should be carefully evaluated in such patients. Additionally, further efforts in terms of developing specific recommendations for influenza vaccination can be explored, where there are no specific guidelines to our knowledge for vaccinations in this subgroup of patients. Lastly, we would like to acknowledge several important limitations. The ability to definitively establish causality is inherently constrained by the observational nature of a single case report, the absence of a rechallenge with brexpiprazole, and the potential influence of confounding variables. Further studies are needed to further elucidate the relationship between brexpiprazole, infectious triggers such as influenza A, and the risk of developing NMS.
